# Carriage and Transmission of *mcr-1* in Salmonella Typhimurium and Its Monophasic 1,4,[5],12:i:- Variants from Diarrheal Outpatients: a 10-Year Genomic Epidemiology in Guangdong, Southern China

**DOI:** 10.1128/spectrum.03119-22

**Published:** 2023-01-11

**Authors:** Ruan-Yang Sun, Liang-Xing Fang, Bi-Xia Ke, Jian Sun, Zuo-Wei Wu, You-Jun Feng, Ya-Hong Liu, Chang-Wen Ke, Xiao-Ping Liao

**Affiliations:** a National Risk Assessment Laboratory for Antimicrobial Resistance of Animal Original Bacteria, South China Agricultural University, Guangzhou, Guangdong, China; b Guangdong Provincial Key Laboratory of Veterinary Pharmaceutics Development and Safety Evaluation, South China Agricultural University, Guangzhou, China; c Guangdong Laboratory for Lingnan Modern Agriculture, Guangzhou, Guangdong, China; d Guangdong Provincial Center for Disease Control and Prevention, Guangzhou, Guangdong, China; e College of Veterinary Medicine, Iowa State University, Ames, Iowa, USA; f Department of Microbiology and Department of General Intensive Care Unit of the Second Affiliated Hospital, Zhejiang University School of Medicine, Hangzhou, Zhejiang, China; g Jiangsu Co-Innovation Center for the Prevention and Control of Important Animal Infectious Diseases and Zoonoses, Yangzhou University, Yangzhou, Jiangsu, China; University of Sydney

**Keywords:** *Salmonella* Typhimurium, colistin resistance, *mcr-1*, genomic epidemiology

## Abstract

The banning of colistin as a feed additive for food-producing animals in mainland China in 2017 caused the decline in the prevalence of Escherichia coli-mobilized colistin resistance (*mcr-1*) in China. Salmonella Typhimurium and its monophasic 1,4,[5],12:i:- variants are also the main species associated with the spread of *mcr-1*; however, the evidence of the prevalence and transmission of *mcr-1* among Salmonella is lacking. Herein, the 5,354 Salmonella isolates recovered from fecal samples of diarrheal patients in Guangdong, Southern China, from 2009 to 2019 were screened for colistin resistance and *mcr-1*, and *mcr-1*-positive isolates were characterized based on whole-genome sequencing (WGS) data. Relatively high prevalence rates of colistin resistance and *mcr-1* (4.05%/4.50%) were identified, and more importantly, the prevalence trends of colistin-resistant and *mcr-1*-positive Salmonella isolates had a similar dynamic profile, i.e., both were first detected in 2012 and rapidly increased during 2013 to 2016, followed by a sharp decrease since 2017. WGS and phylogenetic analysis indicate that, whether before or after the ban, the persistence and cross-hospital transmission of *mcr-1* are primarily determined by IncHI2 plasmids with similar backbones and sequence type 34 (ST34) Salmonella in specific clades that are associated with a high prevalence of IncHI2 plasmids and clinically important antimicrobial resistance genes, including *bla*_CTX-M-14_-*fosA3-oqxAB*-*floR* genotypes. Our work reveals the difference in the prevalence rate of *mcr-1* in clinical Salmonella before and after the Chinese colistin ban, whereas *mcr-1* transmission was closely linked to multidrug-resistant IncHI2 plasmid and ST34 Salmonella across diverse hospitals over 10 years. Continued surveillance is required to explore the factors related to a sharp decrease in *mcr-1* after the recent ban and determine whether the ban has affected the carriage of *mcr-1* in Salmonella circulating in the health care system.

**IMPORTANCE** Colistin is one of the last-line antibiotics for the clinical treatment of *Enterobacteriaceae*. However, the emergence of the mobilized colistin resistance (*mcr-1*) gene has spread throughout the entire human health system and largely threatens the usage of colistin in the clinical setting. In this study, we investigated the existence of *mcr-1* in clinical Salmonella from a 10-year continuous surveillance and genomic study. Overall, the colistin resistance rate and *mcr-1* carriage of Salmonella in tertiary hospitals in Guangdong (2009 to 2019) were relatively high and, importantly, rapidly increased from 2013 to 2016 and significantly decreased after the Chinese colistin withdrawal. However, before or after the ban, the MDR IncHI2 plasmid with a similar backbone and ST34 Salmonella were the main vectors involved in the spread of *mcr-1*. Interestingly, these Chinese *mcr-1*-carrying Salmonella obtain phylogenetically and phylogeographically distinct patterns compared with those from other continents and are frequently associated with clinically important ARGs including the extended-spectrum β-lactamases. Our data confirmed that the national stewardship intervention seems to be successful in blocking antibiotic resistance determinants and that continued surveillance of colistin resistance in clinical settings, farm animals, and related products is necessary.

## INTRODUCTION

Colistin is one of the few “last-resort” antibiotics used for the treatment of critical infections caused by multidrug-resistant (MDR) Gram-negative pathogens, especially carbapenem-resistant *Enterobacteriaceae* (1). Unfortunately, its use has been threatened by the emergence of the mobilized colistin resistance gene (*mcr-1*), first discovered in China in 2016 (2) but now disseminated across the world. MCR genes encode phosphoethanolamine transferase that modifies lipid A to block the binding of polymyxin antibiotics and confers low levels of colistin resistance. To date, MCR family genes involved other nine distinct variants (*mcr-2* to *mcr-10*), but the degree of similarity in amino acid sequences between them is variable, from 31.0% to 83.0% amino acid identity with *mcr-1* (3). Compared with other *mcr* varients, *mcr-1* is the most prevalent of mobile colistin resistance genes and has been identified in a variety of bacterial species, primarily Escherichia coli of diverse ecological niches, including animals, humans, and environments (3–5). Furthermore, *mcr-1* has a global presence in MDR Salmonella, in particular sequence type 34 (ST34) Salmonella Typhimurium and its monophasic variant Salmonella enterica subsp. serotype 1,4,[5],12:i:-, from not only food animals and animal-derived food products but also humans, including hospital patients with nosocomial infections (6–8). A complex transposon Tn*6330* containing *mcr-1* (1,626 bp) and a PAP2 superfamily protein-encoding gene (765 bp), and IS*Apl1* on both sides, was proposed to be the key element mediating translocation of *mcr-1* into various plasmid backbones (4). However, Tn*6330* was not stable, and IS*Apl1* whose location is downstream of *mcr-1* is not as stable as it is in the upstream of *mcr-1* (9). To date, various types of plasmids (58 to 251 kbp) are involved in *mcr-1* dissemination among human-infected isolates, with IncI2, IncX4, and IncHI2 being the most predominant plasmid types (10).

Given colistin's minimal use in clinical use, in particular salmonellosis (11), *mcr-1* of clinical Salmonella origin is most likely linked to food animals (12). The withdrawal of colistin sulfate premix as a prophylactic growth promoter in animal feed was approved in mainland China in April 2017, resulting in a rapid, ecosystem-wide decline in the presence of the *mcr-1* gene. This was observed not only in animals but also in human feces and infections (13, 14). However, both studies focused primarily on the E. coli *mcr-1* host and did not consider other closely related *Enterobacteriaceae* species such as Salmonella. As described above, besides E. coli, Salmonella was also a significant reservoir for *mcr-1*, although little information is available on the prevalence of *mcr-1* in Salmonella after the postintervention period. Typhimurium is one of the common serovars, and it is considered as more MDR than other serovars and the main serovar harboring *mcr-1* (15). With time, several variants of *S.* Typhimurium, particularly a monophasic variant of Typhimurium, have become dominant within animal and human samples in the past two decades (16, 17). Here, we provide a retrospective analysis of the prevalence of colistin resistance and the presence of the *mcr* gene in clinical Salmonella isolates, in particular Typhimurium and its monophasic variants from 2009 to 2019. Together with whole-genome sequencing (WGS), we used phylogenetic analysis to describe these *mcr-1*-positive isolates.

## RESULTS

### Prevalence of *mcr-1* in Salmonella Typhimurium and its monovarient.

**FIG 1:**
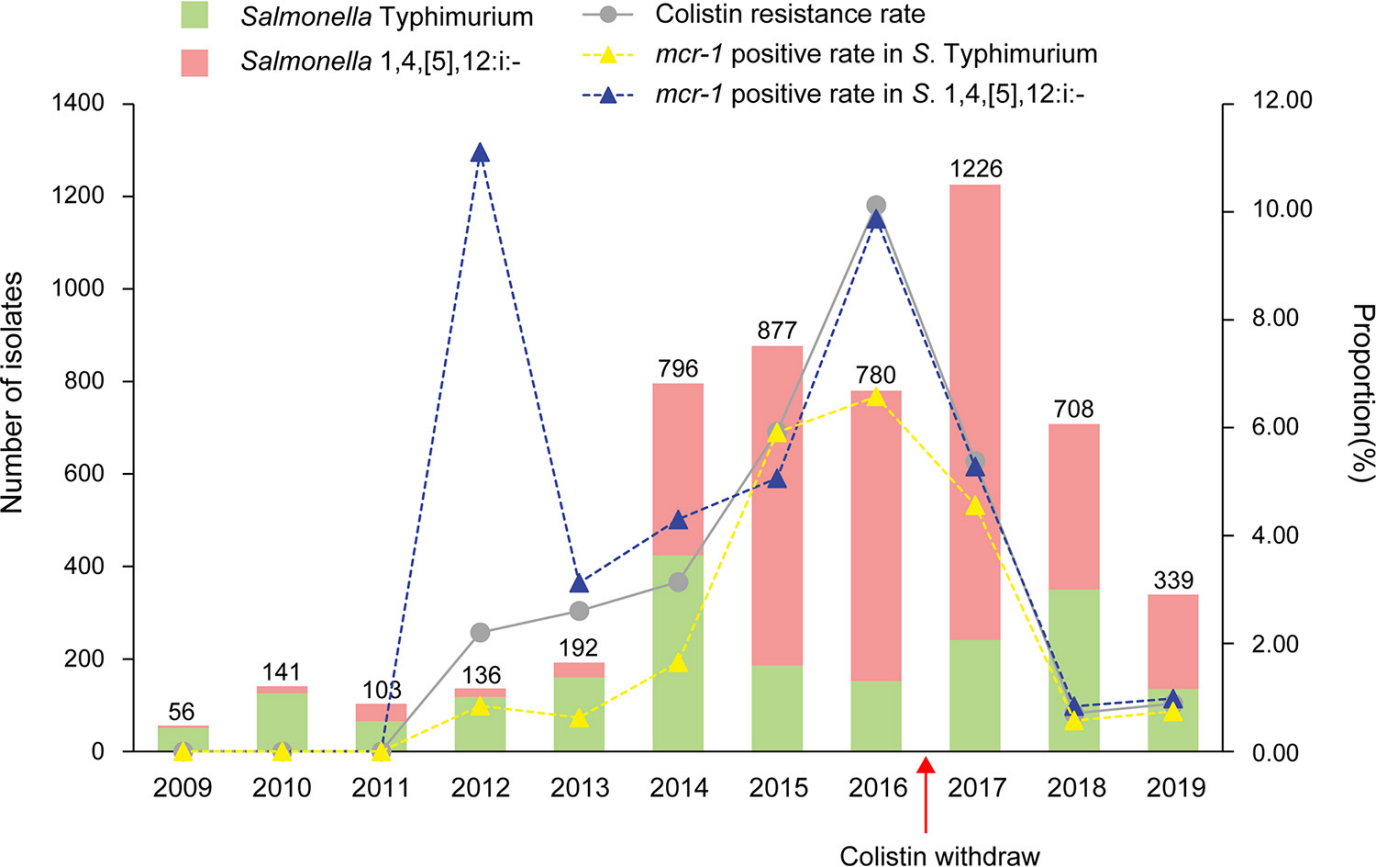
Proportion of colistin resistance and *mcr-1* in Salmonella isolates from diarrheal patients, Guangdong, China.

This study was comprised of 5,354 *S.* Typhimurium and its monophasic *S.* 1,4,[5],12:i:- isolates, of which 238 (4.45%) were resistant to colistin phenotypically. Genotypically, 230 (4.30%) possessed the *mcr* gene, including 217 (4.05%) containing *mcr-1*, 12 (0.22%) with *mcr-3* as previously described (18), and a single (0.2%) *mcr-9*-positive isolate. Additionally, of the 217 *mcr-1*-carrying Salmonella isolates 44 (44/2,008, 2.19%) were *S.* Typhimurium and 173 (173/3,346, 5.17%) were *S*. 1,4,[5],12:i:- (Table S2 in the supplemental material). All 230 *mcr*-positive Salmonella isolates were colistin resistant in the range of 4 to 16 mg/L. The prevalence of colistin-resistant and *mcr-1*-positive Salmonella isolates in each serotype showed a similar trend: both were first detected in 2012 and remained low in 2013 and 2014 (1.04% to 3.14%). A rapid increase occurred between 2015 and 2016 (5.25% to 10.13%) followed by a sharp decrease starting in 2017 that reached a minimum (<1%) between 2018 and 2019 (Fig. 1). The *mcr-1*-positive rates in monophasic 1,4,[5],12:i:- were generally higher than Typhimurium in the last decade. Additionally, the *mcr-1* gene was identified in 24 hospitals in 11 cities in Guangdong. Heyuan displayed the highest *mcr-1* prevalence (9.62%) and Guangzhou the lowest (2.06%) (Fig. S1).

### Antibiotic resistance profiles of *mcr-1*-carrying isolates.

Antimicrobial susceptibility testing indicated that the majority of 217 *mcr-1*-positive Salmonella isolates were resistant to doxycycline (96.31%), chloramphenicol (94.47%), ampicillin (92.63%), florfenicol (92.17%), sulfamethoxazole/trimethoprim (85.71%), cefotaxime (82.03%), gentamicin (78.80%), and fosfomycin (73.27%). In contrast, a relatively smaller number of *mcr-1*-positive isolates were resistant to amikacin (41.01%), ciprofloxacin (40.09%), and ceftazidime (15.21%). All isolates were susceptible to meropenem and tigecycline (Table S3). Generally, most antimicrobial resistance rates were increased in the postintervention period such as cefotaxime, fosfomycin, ampicillin, chloramphenicol, and gentamicin, while others were not changed significantly (Fig. S2). For different serovars, resistance to chloramphenicol, amikacin, and fosfomycin was significantly higher among 1,4,[5],12:i:- isolates than Typhimurium (*P < *0.05), while other antibiotics showed no significant difference (*P > *0.05) (Fig. S3).

A total of 127 *mcr-1*-positive Salmonella isolates were selectively sequenced (including all 36 *mcr-1*-positive isolates from 2012 to 2014 and 2018 to 2019, and 91 randomly picked isolates from 2015 to 2017). The genomes of these isolates possessed a median number of 19 antimicrobial resistance genes (ARGs) (ranging from 4 to 26). Of note, there was no significant difference in the average number of ARGs among these 127 *mcr-1*-positive Salmonella isolates before and after the ban (17.4 from 2012 to 2014, 19.6 from 2015 to 2016, and 16.7 from 2017 to 2019) (Fig. S4). In addition to *mcr-1*, other clinically important ARGs were also highly prevalent and these included the extended-spectrum β-lactamase (ESBL) gene *bla*_CTX-M-14_ (60.6%, 77/127), the fosfomycin resistance gene *fosA3* (64.6%, 82/127), the plasmid-mediated quinolone resistance gene *oqxAB* (78.7%, 100/127), and the phenicol resistance gene *floR* (89.8%, 114/127). Furthermore, a number of other ARGs were also present at a prevalence rate >50%, including *aac(6’)-Iaa*, *aac(3)-IVa*, *strAB*, *aph(4)-Ia*, *aphA1*, *ant(3′')-Ia*, *aadA2*, *bla*_TEM_, *cmlA1*, *sul1/2/3*, *dfrA12*, and *tet*(B). Among these 127 *mcr-1*-positive isolates, a total of 19 distinct plasmid replicon types was found, with IncHI2 being the most prevalent (92.9%, 118/127), followed by IncX4 (15.7%, 20/127) and IncN (10.2%, 13/127). Of note, the replicon IncHI2 type coexisted more with the ARGs presenting a prevalence rate >50%, including *bla*_CTX-M-14_-*fosA3*-*oqxAB*-*floR* (namely, CFOF genotype).

### *mcr-1*-positive plasmid analysis.

**FIG 2:**
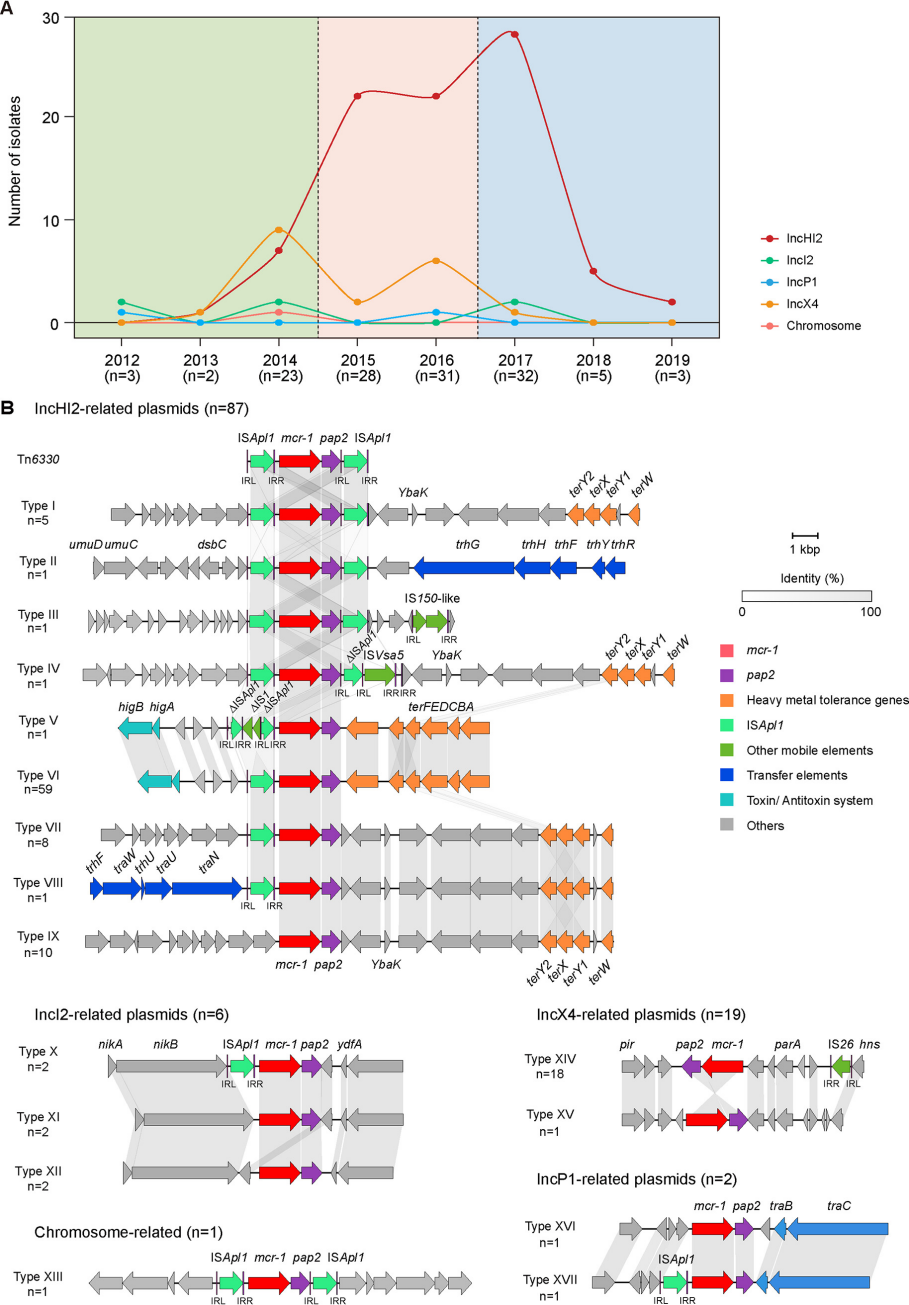
Genetic structures of plasmids and chromosomes from *mcr-1*-positive Salmonella isolates. (A) Proportional dynamics of the genetic context of *mcr-1* in 115 sequenced Salmonella isolates by designated period. (B) Genetic environments of *mcr-1* gene in bacterial plasmids and chromosomes.

The combination of BLAST analysis and mapping using the WGS data enabled us to assign *mcr-1* locations in 115 of the 127 *mcr-1*-positive Salmonella isolates, including 114 plasmid borne and 1 chromosome borne. IncHI2 plasmids (87/115, 75.65%) were the most prevalent while IncX4 (19/115, 16.52%), IncI2 (6/115, 5.22%), and IncP1 (2/115, 1.74%) formed smaller subsets. IncX4 plasmid was the predominant location for *mcr-1* in the 2012 to 2014 period. After 2015, the IncHI2 plasmid replaced the IncX4 plasmid and become the dominant *mcr-1* plasmid type over 5 years (2015 to 2019), indicative of a stable IncHI2 group distribution. However, the proportion of other plasmid-associated *mcr-1*-positive Salmonella continuously decreased in postintervention (2017 to 2019) periods (Fig. 2A). Conjugation assay showed that the *mcr-1* gene and colistin resistance phenotypes were transferable among 67 of 127 sequenced *mcr-1*-positive isolates. This indicated that transferable *mcr-1*-carrying plasmid types of IncHI2 (46/87, 52.87%), IncI2 (4/6, 66.67%), IncX4 (11/19, 57.89%), and IncP1 plasmids (2/2, 100%) were present in our Salmonella isolates.

Based on the different insertion sites of the mobile elements, the genetic environments of *mcr-1* among these 127 sequenced isolates were clustered into 23 types, including 9 for IncHI2, 3 for IncI2, 2 for IncX4, 2 for IncP1, 1 type in the chromosome, and 6 in isolates where the *mcr-1* location was not determined (Fig. 2B; Fig. S5A). Among 87 *mcr-1*-carrying IncHI2 plasmids, the genetic environments of *mcr-1* were diverse and inserted into several sites of the IncHI2 plasmid backbone. Tn*6330*, characterized by the structure IS*Apl1*-*mcr-1*-*pap2*-IS*Apl1*, was shown to be responsible for the dissemination of *mcr-1*, and the *mcr-1*-carrying fragments of different sizes in this study shared 100% nucleotide sequence identity with the corresponding region of Tn*6330*. Complete Tn*6330* and partially truncated Tn*6330* were found in only eight strains. In contrast, one copy of IS*Apl1* upstream of *mcr-1* (*n* = 68) and the absence of IS*Apl1* (*n* = 10) were far more common in the collection. Type VI was the most prevalent type (*n* = 59) where IS*Apl1* was located upstream and the tellurium resistance genes cluster *terABCDEF* was downstream of *mcr-1* (Fig. 2B). This likely represents a single IS*Apl1*–*mcr-1*–IS*Apl1* acquisition and subsequent loss of IS*Apl1*, indicating the downstream IS*Apl1* was not stable. In addition, We also observed truncated IS*1* appear upstream of the *mcr-1* gene and inserted into the IS*Apl1* (type V), resulting in the deletion of the IS*Apl1*.

We obtained 14 complete *mcr-1*-carrying IncHI2 plasmid sequences (142.938 to 258.199 kbp) using a combination of Illumina and ONT sequencing. Similarly, the 14 large *mcr-1*-carrying IncHI2 plasmids showed an extremely high similarity to the first reported *mcr-1*-carrying IncHI2 plasmid pHNSHP45-2 and possessed the prototypical IncHI2-type backbone. The core functional genes, including those responsible for plasmid replication, conjugal transfer, maintenance, and stability, were found in all the plasmids, except for four that lacked conjugative transfer region I composed of *traJGIH* and *trhRYXFHG* genes. MDR regions located on IncHI2 plasmids likely evolved through the recombination and integration of a variety of ARGs, such as *bla*_CTX-M-14_, *oqxAB*, *aac(6′)-Ib-cr*, *qnrS1*, *floR*, and *fosA3*, with the help of mobile genetic elements, especially IS*26* (Fig. S6). The insertion of *mcr-1* occurred in the backbone randomly while other ARGs were located in the variable region, indicating the *mcr-1* gene was captured by plasmids independently. Furthermore, not only *mcr-1* but also *mcr-9* was located on the IncHI2 plasmid. *mcr-9* was bracketed by two complete insertion sequences (IS*903* and IS*26*) in its flanking regions on 307.309-kbp IncHI2 plasmid. The *mcr-9*-carrying IncHI2 plasmid showed high similarity to backbone elements of IncHI2 on a large scale, suggesting that IncHI2 plasmid served as the main vehicle in carrying MCR family genes, especially *mcr-1* and *mcr-9* in Salmonella (Fig. S7).

Unlike *mcr-1*-carrying IncHI2 plasmids, the genetic environments of *mcr-1* in the other *mcr-1*-carrying small-size plasmid were relatively conserved. We obtained 24 complete *mcr-1*-carrying small-size plasmid sequences (<100 kb) using a combination of Illumina and Sanger sequencing. These were represented by 16 IncX4 (31.778 to 34.645 kbp), 6 IncI2 (57.627 to 60.346 kbp), and 2 IncP1 (48.944 to 49.298 kbp) replicons. Circularization of the plasmid sequences indicated that the backbones of the small *mcr-1*-carrying plasmids (IncX4, IncI2, and IncP1) were highly conserved compared to their corresponding prototypical plasmid types while the variable regions lacked any other ARGs but *mcr-1* (Fig. S5B to D). IS*Apl1* can be either present or absent in IncI2-type plasmids and IncP1-type plasmids and completely absent in IncX4-type plasmids (~30 kb). However, a complete insertion sequence, IS*26*, was found ~3.5 kbp upstream of *mcr-1* in the IncX4 plasmid, suggesting a unique pattern in the *mcr-1* acquisition. The only chromosomally encoded *mcr-1* strain 14E1005 carried composite Tn*6330*, indicating transposition-mediated chromosome insertion.

### Phylogeny of *mcr-1*-producing Salmonella.

Among these 127 *mcr-1*-positive isolates, multilocus sequence typing (MLST) analyses indicated the presence of six kinds of STs; ST34 (*n* = 104) and ST34-SLV (single-locus variant) (*n* = 10) were the most prevalent (89.8%, 114/127) followed by ST19 (*n* = 6), ST5401 (*n* = 4), ST36, ST6020, and ST8067 (*n* = 1). We undertook phylogenomic analyses of 127 isolates from the current study as well as 13 isolates from livestock in our WGS collection, contextualized with 112 publicly available isolates from multiple geographical regions. The complete list of analyzed genomes with information, including isolation year, country, and source, is presented in Table S4.

**FIG 3:**
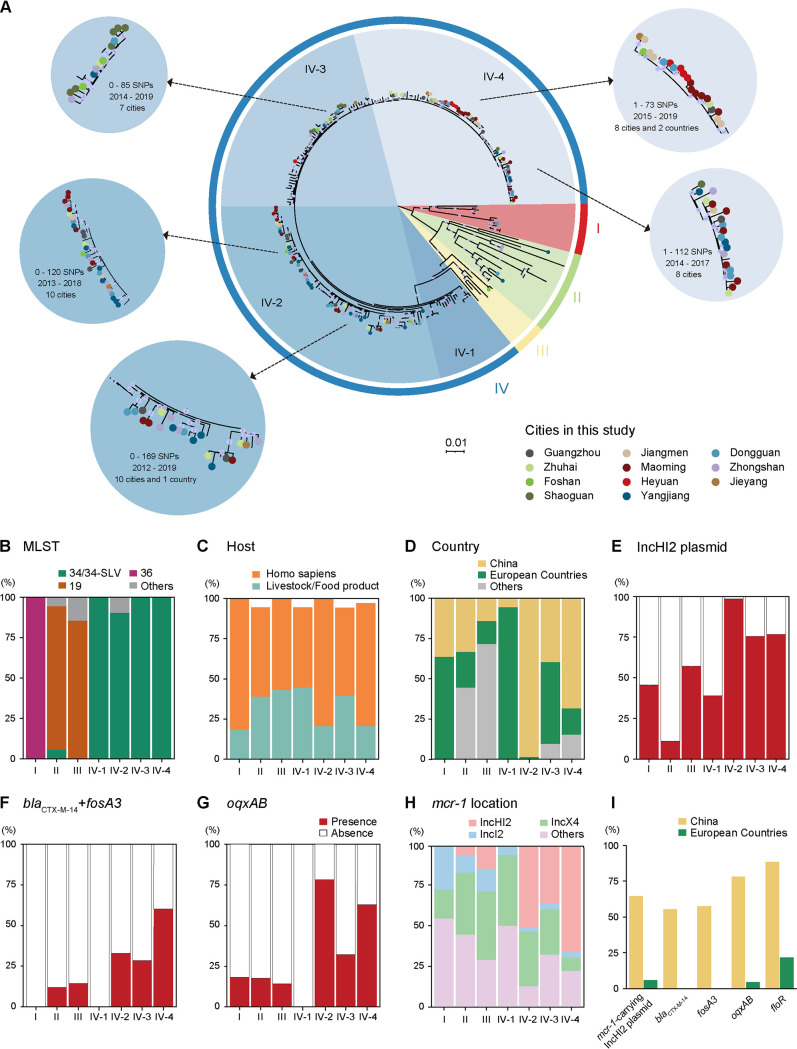
Phylogenetic analysis of the *mcr-1*-positive Salmonella, including 127 isolates from this study (different colors represent the sites of sampling), 13 isolates from animal-originated samples, and 112 global isolates in NCBI database. (A) Maximum likelihood phylogeny of *mcr-1*-positive Salmonella isolates from this study in the context of other global *mcr-1*-positive isolates based on 8,868 nonrecombinant SNPs. The genome of Salmonella Typhimurium strain LT2 was used as reference. Internal nodes are labeled with circles indicating branch >70% bootstrap support. (B to H) Frequency distribution of MLST (B), host (C), country (D), IncHI2 plasmid (E), *bla*_CTX-M-14_ combined with *fosA3* (F), *oqxAB* (G), and *mcr-1* location (H) in each clade and subclade. (I) Pairwise comparison of *mcr-1*-positive IncHI2 plasmid and CFOF genotypes within China and European countries.

A maximum likelihood (ML) tree was constructed on basis of 8,868 nonrecombinant single nucleotide polymorphism (SNPs) obtained from the core-genomes of 252 *mcr-1*-positive *S.* Typhimurium and monophasic variants 1,4,[5],12:i:- (Fig. 3A). Phylogenetic analyses indicated a low nucleotide divergence across the core chromosomal genes with a median pairwise distance of 108 SNPs with a range of 0 to 1,053 SNPs (Fig. S8A). The hierarchical Bayesian clustering analysis divided all 253 isolates into 4 distinct clades, and compared to other clades, isolates in clade IV included 4 subclades, IV-1 to IV-4, and it was highly related with a median pairwise core SNP distance of 92 (interquartile range, 0 to 214; Fig. S8B and Table S5). Notably, clades I to III (*n* = 36) included primarily ST19/36 types and clade IV (*n* = 217) included primarily ST34 (Fig. 3B). Each clade and subclade contained both human and livestock/food isolates with no obvious correlation between clades, subclades, and host (Fig. 3C).

More importantly, each clade and subclade contained both Chinese and European isolates, but, of note, clade IV-2 (98.6%, 72/73) and IV-4 (68.5%, 50/73) were mainly composed of Chinese isolates. By comparison, isolates from Europe were primarily clustered in clade I (63.6%, 7/11) and clade IV-1 (94.4%, 17/18). Clade IV-3 was mainly composed of both European (50.9%, 27/53) and Chinese (39.6%, 21/53) isolates (Fig. 3D; Table S6). It is worth noting that *mcr-1*-positive isolates from different cities in the Guangdong province cluster together in each of the subclades of clade IV. Furthermore, isolates from the different hospitals in different cities often formed branches with high similarity (0 to 169 SNPs) over 2012 to 2019, indicating the clonal spread of Salmonella occurred and circulated in Guangdong (Fig. 3A). Compared to clades I, II, III, and IV-1, clades IV-2, IV-3, and IV-4 were more prevalent in association with IncHI2 replicon (>75.5% versus 30.6/38.9%) and clinically significant ARGs, *bla*_CTX-M-14_, *fosA3*, and *oqxAB* (30.2 to 78.1 versus 8.3 to 16.7/0 to 5.6%) (Fig. 3E to G). Consistently, *mcr-1*-positive IncHI2 plasmids were more prevalent in clades IV-2, IV-3, and IV-4, while *mcr-1*-positive IncX4 plasmids were more prevalent in clades II, III, and IV-1 (Fig. 3H). Furthermore, intriguingly, the *mcr-1*-carrying IncHI2 plasmid as well as the CFOF genotype was much more prevalent in Chinese ST34 *mcr-1*-positive Salmonella isolates than in European isolates (Fig. 3I).

**FIG 4:**
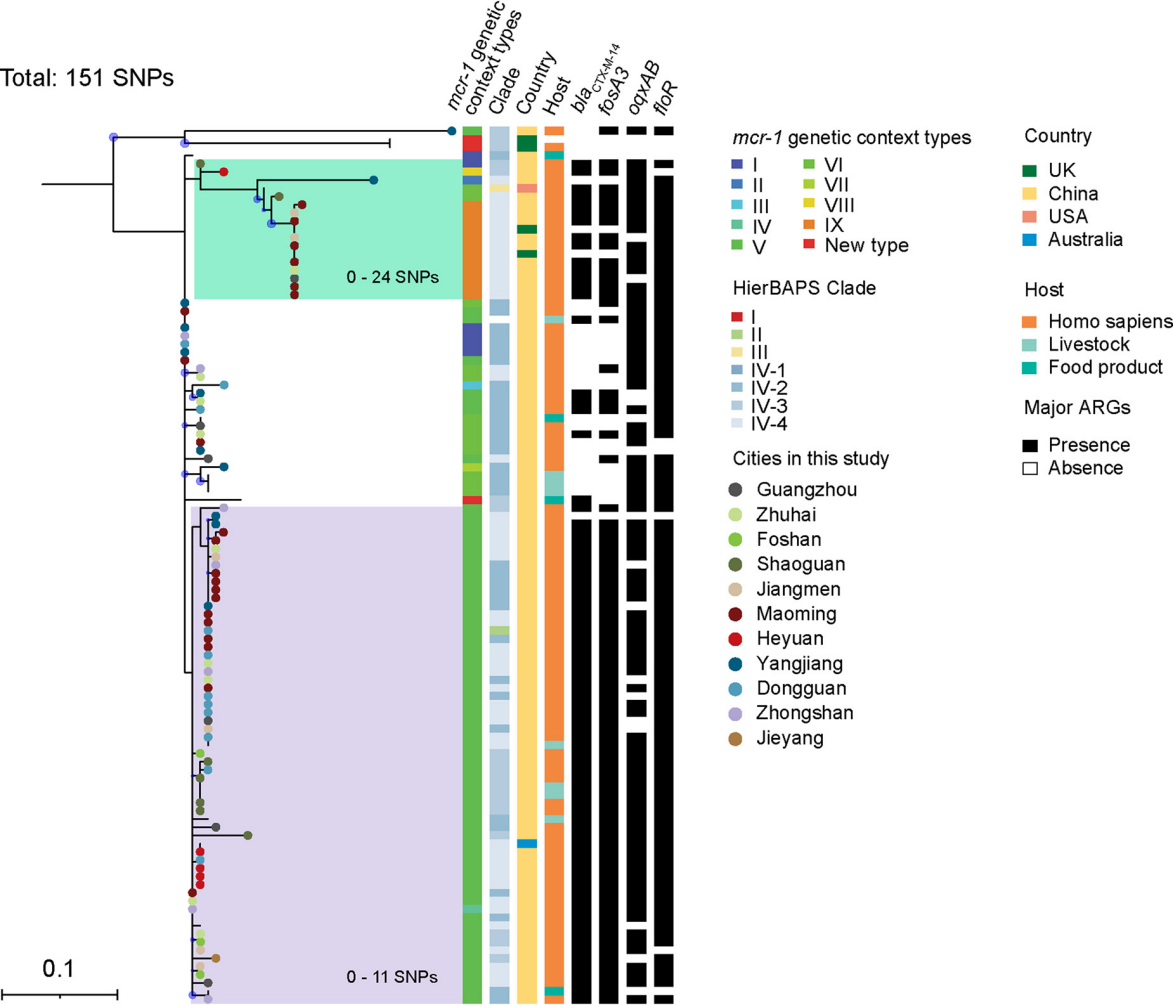
Maximum-likelihood phylogeny of 106 isolates carrying *mcr-1*-positive IncHI2 plasmids in comparison with the pHNSHP45-2 plasmid (accession no. KU341381). Internal nodes are labeled with circles indicating branch >70% bootstrap support.

To determine the genetic relatedness of *mcr-1*-positive IncHI2 plasmids, all 106 isolates carrying *mcr-1-*positive IncHI2 plasmids were selected, including 87 from humans, 6 from livestock in this study, and 13 from publicly available genomes. These 106 isolates were distributed among all of the hierarchical Bayesian Analysis of Population Structure (HierBAPS) clades as described above but clade I. We constructed phylogenetic trees based on 151 SNPs on the nucleotide composition of a set of IncHI2 plasmid core genes (Fig. 4; Table S7), indicating a highly conserved *mcr-1* bearing the IncHI2 plasmid backbone structure had spread in China, particularly different cities among Guangdong province, as well as other countries, including the United Kingdom, United States, and Australia. However, the *mcr-1* genetic context distribution was highly diverse. In addition, most Chinese Salmonella isolates bearing *mcr-1-*positive IncHI2 plasmids possessed the CFOF genotype. In contrast, almost all European ones did not carry *bla*_CTX-M-14_-*fosA3*.

## DISCUSSION

We performed a 10-year continuous surveillance study of the prevalence of colistin resistance and *mcr-1* among *S.* Typhimurium and its monophasic *S.* 1,4,[5],12:i:- isolates from a large collection of diarrheal outpatients samples from Guangdong province, Southern China. Our results indicated that the prevalence of *mcr-1* (4.05%) was much higher in our tested clinical Salmonella isolates, in particular during 2015 to 2017 (5.25 to 9.23%), than that previously reported for *mcr-1* in Salmonella isolates from humans and animals origins (6, 7, 19). Published data have reported the prevalence of *mcr-1* is low in clinical Salmonella derived from infectious diseases (~1 to 2%) (12, 20). The main reason for the high prevalence of *mcr-1* in the current study is that the majority of the sampled Salmonella was the newly emerging monophasic variant *S*. 1,4,[5],12:i:-, mostly belonging to ST34 (21). The key feature of this serovar along with Typhimurium is the increasingly extensive MDR. The *mcr-1*-positive Salmonella was found to frequently coexist with multiple other ARGs, including ESBLs and plasmid-mediated quinolone resistance (PMQR), and *mcr-1* was mostly located on MDR IncHI2 plasmids. The usage of ESBL drugs and fluoroquinolones in a clinical setting would coselect the *mcr-1*-carrying Salmonella. It may, to some extent, explain the high *mcr-1* prevalence in this study. Furthermore, the sharp increase of *mcr-1* from 2015 to 2017 suggests that *mcr-1* was rapidly spreading in clinical settings, potentially attributable to an extremely high *mcr-1* prevalence (>60%) in livestock around the time (22). Similarly, the prevalent trend of colistin-resistance and *mcr-1*-positive Salmonella isolates dramatically fell during 2018-19, and it was consistent with that in animal-derived E. coli isolates in China documented in another report (23), which was due to the direct consequence of the ban of colistin as an animal feed additive in China after April 2017. It is still necessary to determine whether the Chinese national stewardship intervention led to keeping *mcr-1*-positive and colistin-resistant Salmonella out of the human health system.

We showed that both clonal and horizontal transmission contributed to *mcr-1* dissemination across Guangdong, even in China. Chinese *mcr-1*-positive ST34 isolates mainly distributed clade IV, and the cgSNPs of ST34 Salmonella clone in each subclade IV, in particular IV-2, IV-3, and IV-4, remained low even in diverse cities and different hosts in the Guangdong province, suggesting that the clonal transmission happened frequently and responded to the spread of *mcr-1* among Salmonella isolates during the last decade, whether or not colistin was bannned as an animal food additive. Furthermore, intriguingly, Chinese *mcr-1*-positive ST34 isolates mainly distributed in clade IV-2 and IV-4 were associated with a high prevalence of the CFOF genotype and *mcr-1*-positive IncHI2 plasmids. IncHI2 plasmids are typically large (>250 kb) with a wide host range. The WGS analysis of *mcr-1*-positive Salmonella isolates and *mcr-1*-bearing IncHI2 plasmids indicated that the acquisition of MDR IncHI2 plasmids probably accounts for the coexistence of *mcr-1* with the CFOF (*bla*_CTX-M-14_-*fosA3*-*oqxAB*-*floR*) genotype in the ST34 isolates from this study. This is consistent with previous epidemiological studies that IncHI2 plasmids are likely to be the major vectors for cospreading the clinically important ARGs, including *bla*_CTX-M_, *oqxAB*, *mcr-1*, *fosA3*, and *floR* in China (24, 25).

Of note, the Chinese *mcr-1*-carrying IncHI2 plasmids also clustered together with some from the United Kingdom, United States, and Australia. Although >50% *mcr-1*-positive ST34 from Europe also possessed IncHI2 plasmids, these European IncHI2 plasmids commonly carried no CFOF genotype except for *floR*, which is consistent with European *mcr-1*-positve Salmonella without the CFOF genotype. Commonly, prevalent AMR clones emerge in response to local antimicrobial usage and undergo population expansion under selection from sustained antimicrobial exposure (26). According to the European Medicines Agency, the third- and fourth-generation cephalosporins, fluoroquinolones, other quinolones, and polymyxins only make up 0.2%, 2.5%, 0.3%, and 3.3% of total antibiotic consumption, which were much lower percentages than those in China. Furthermore, current epidemiological studies indicate a relatively high prevalence of *bla*_CTX-M_, *oqxAB*, *fosA3*, and *floR* in Enterobacteriaceae, in particular E. coli, *S.* Typhimurium, and *S*. Indiana from both food-producing animals and humans in China (7, 27). Thus, we speculate that capturing local epidemic ARGs by IncHI2 plasmids resulted in Chinese *mcr-1*-positive ST34 isolates associated with the CFOF genotype and that this might, to some extent, explain why the CFOF genotype frequently occurred in the *mcr-1*-carrying IncHI2 plasmids among ST34 Salmonella isolates from China and not from Europe.

Taken together, the emergence, spread, and dispersion of *mcr-1*-positive ST34 Salmonella isolates in China, particularly in the Guangdong province during the past decades, were probably closely related to antibiotic consumption and geography. Of note, during the last decade, including before and after the Chinese colistin withdrawal, these Chinese *mcr-1*-carrying IncHI2 plasmids in our analysis shared similar backbones comprising the replicon, the partition system, the conjugative apparatus, and the telluric resistance cluster, implying the circulation of epidemic MDR *mcr-1*-carrying IncHI2 plasmids in clonal ST34 Salmonella isolates across China, in particular hospitals in Guangdong province. Some of the plasmid-bacterium associations become particularly successful, such as Klebsiella pneumoniae ST11 or ST405 and plasmid pOXA-48, E. coli ST131 clade C2/H30Rx, and IncFII plasmids carrying *bla*_CTX-M_, creating “superbugs” that disseminate uncontrollably in clinical settings (28). Further study is required to determine whether the epidemic MDR IncHI2 plasmids still maintain in *S.* Typhimurium and its monophasic *S.* 1,4,[5],12:i:- ST34 Salmonella clones after the Chinese ban.

### Conclusions.

This work represents the first 10-year continuous surveillance and genomic study of *mcr-1*-positive Salmonella isolates from Guangdong, Southern China, and it provides the prevalence trends of colistin resistance and *mcr-1* in clinical Salmonella isolates before and after the Chinese colistin withdrawal. The prevalence trends of colistin-resistant and *mcr-1*-positive Salmonella isolates have a similar dynamic profile, i.e., both were first rapidly increased from 2013 to 2016, followed by a sharp decrease since 2017. However, before and after the ban, we found that both the persistence and spread of *mcr-1* across diverse hospitals and cities in Guangdong over 10 years were primarily attributed to MDR IncHI2 plasmids with similar backbones and ST34 Salmonella in specific clades associated with a high prevalence of IncHI2 plasmids and clinically important ARGs, including *bla*_CTX-M-14_-*fosA3-oqxAB*-*floR* genotypes. Continued surveillance is required to explore the factors related to a sharp decrease in *mcr-1* in our studies after the recent ban and determine whether the ban has affected the carriage of *mcr-1* in Salmonella circulating in the health care system.

## MATERIALS AND METHODS

### Bacterial strains, screening for *mcr* genes, and antimicrobial susceptibility testing.

Nonrepeated Salmonella isolates (*n* = 5,354) including 2,008 Salmonella Typhimurium and 3,346 Salmonella 1,4,[5],12:i:- were recovered from 2009 to 2019 from 46 hospitals in 14 of 21 cities of Guangdong province, China (Table S1). These isolates were collected by the Guangdong Provincial CDC from outpatients with diarrhea through a clinic-based Salmonella infection surveillance program (18). All 5,354 Salmonella isolates were subjected to antimicrobial susceptibility testing for colistin resistance by broth dilution, and the resistance breakpoint was interpreted according to the EUCAST v 12.0 (>2 mg/L). Simultaneously, all isolates were screened for the presence of *mcr-1* to *mcr-9* using the PCR as previously described (2). Antimicrobial susceptibility testing of *mcr*-positive isolates against ampicillin, cefotaxime, ceftazidime, meropenem, ciprofloxacin, gentamicin, amikacin, florfenicol, doxycycline, tigecycline, fosfomycin, and sulfamethoxazole/trimethoprim was performed by agar diffusion according to CLSI except for tigecycline where the broth dilution method was used (29, 30). E. coli ATCC 25922 served as the quality control strain. The results were interpreted according to CLSI 28th edition instructions while tigecycline resistance was defined according to EUCAST v 12.0 clinical breakpoints.

### Genome sequencing and data analysis.

The total genomic DNA of *mcr-1*-positive isolates was extracted, and DNA libraries were constructed with 250-bp paired-end fragments and sequenced using the Illumina HiSeq system (San Diego, CA, USA). The sequence reads were assembled into contigs using SPAdes v3.13.0 (31). To obtain the complete genome sequence, randomly selected isolates harboring *mcr-1*-positive IncHI2 plasmids were further selected for long-read sequencing (Oxford Nanopore, Oxford, UK). *De novo* hybrid assembly using short and long reads was performed using Unicycler v0.4.4 (32, 33). Sequencing quality and statistics per isolates were checked using the QualiMap v2.2.2 (34). ARG profiling and plasmid replicon typing were performed with ABRicate v1.0.1 (https://github.com/tseemann/abricate) using ResFinder v4.1 (35) and PlasmidFinder v2.1 (36) as reference databases. *In silico* Salmonella serotyping and MLST were performed by SISTR (37) and MLST v2.19.0 (https://github.com/tseemann/mlst), respectively.

### Gene location, genetic environment, and transfer of *mcr-1* analysis.

Contigs harboring *mcr-1* were searched and extracted from the assemblies using contig-puller (https://github.com/kwongj/contig-puller). PCR and Sanger sequencing was used to close all sequence gaps as appropriate. All assemblies were annotated using Prokka v1.14.6 (38). Insertion sequences were identified using ISfinder (39). By searching against PlasmidFinder (>95% identity and >90% coverage), *mcr-1*-carrying contigs harboring plasmid replicons were identified and considered plasmid borne. Comparisons of genetic contexts surrounding the *mcr-1* gene were undertaken using Clinker v0.0.25 and BRIG v0.95 (40, 41). To determine the transferability of *mcr-1*, conjugation experiments were performed by a liquid mating-out assay using streptomycin-resistant E. coli C600 as the recipient strain as previously described (42). Antimicrobial susceptibility testing was conducted on transconjugants, and the transfer of *mcr-1* was confirmed using PCR.

### Phylogenetic analysis.

To identify SNP sites, the assembled reads of *mcr-1*-positive Salmonella isolates were mapped to the reference sequence of *S.* Typhimurium LT2 (accession no. AE006468.2) using Snippy v4.4 (https://github.com/tseemann/snippy). Recombinant regions were excluded using Gubbins v2.4.1 (43). The ML tree was then computed with RAxML v8.2.4 (GTRGAMMA substitution model) (44) with 100 bootstrap replicates to assess support and the corresponding features of each isolate were visualized using iTOL v4 (45). The population structure was investigated using HierBAPS (46). Assembled reads from isolates containing *mcr-1*-bearing IncHI2 plasmids were mapped to pHNSHP45-2 (accession no. KU341381) as a reference plasmid using Snippy as described above. The resultant multiple sequence alignment composed of 151 SNPs was used to infer a maximum-likelihood phylogeny using RAxML.

### Statistics analysis.

Data were collected in Excel (Microsoft; https://www.microsoft.com) and processed and analyzed with Microsoft Office Excel and R (https://www.r-project.org/) thereafter. Statistical significance (*P* values) of normalized data was determined by R using the χ^2^ test.

### Data availability.

All WGS data obtained in this study are available on NCBI with BioProject ID: PRJNA629650.
