# Supplementary material for: Carriage and Transmission of mcr-1 in Salmonella Typhimurium and Its Monophasic 1,4,[5],12:i:- Variants from Diarrheal Outpatients: a 10-Year Genomic Epidemiology in Guangdong, Southern China
Source: Microbiol Spectr. 2023 Jan 11;11(1):e03119-22. doi: 10.1128/spectrum.03119-22 (PMC9927551; doi:10.1128/spectrum.03119-22)
Supplement: Supplemental file 1 — Supplemental material. Download spectrum.03119-22-s0001.pdf, PDF file, 2.4 MB [file spectrum.03119-22-s0001.pdf]

Supplemental materials for

Carriage and transmission of *mcr-1* in *Salmonella* Typhimurium and its monophasic 1,4,[5],12:i:- variants from diarrhoeal outpatients: a 10-year genomic epidemiology in Guangdong, Southern China

Ruan-Yang Sun[a,b*], Liang-Xing Fang[a,b,c*], Bi-Xia Ke[d], Jian Sun[a,b,c], Zuo-Wei Wu[e], You-Jun Feng[f], Ya-Hong Liu[a,b,c,g], Chang-Wen Ke[d], Xiao-Ping Liao[a,b,c#]

[a]National Risk Assessment Laboratory for Antimicrobial Resistance of Animal Original Bacteria, South China Agricultural University, Guangzhou, Guangdong, China;

[b]Guangdong Provincial Key Laboratory of Veterinary Pharmaceutics Development and Safety Evaluation, South China Agricultural University, Guangzhou, China;

[c]Guangdong Laboratory for Lingnan Modern Agriculture, Guangzhou, Guangdong, China;

[d]Guangdong Provincial Center for Disease Control and Prevention, Guangzhou, Guangdong, China;

[e]College of Veterinary Medicine, Iowa State University, Ames, Iowa, USA;

[f]Department of Microbiology and Department of General Intensive Care Unit of the Second Affiliated Hospital, Zhejiang University School of Medicine, Hangzhou, Zhejiang, China;

[g]Jiangsu Co-Innovation Center for the Prevention and Control of Important Animal Infectious Diseases and Zoonoses, Yangzhou University, Yangzhou, Jiangsu, China.

* These authors contributed equally.

[#] Corresponding author: Xiao-Ping Liao (xpliao@scau.edu.cn).

Supplemental tables

Table S1. Metadata of 5354 clinical *Salmonella* isolates in this study

Table S2. Comprehensive information of 217 *mcr-1*-positive *Salmonella* isolates in this study

Table S3. Antimicrobial susceptibility of 217 *mcr-1*-positive *Salmonella* Typhimurium and 1,4,[5],12:i:- and 118 transconjugants (MICs, mg/L)

Table S4. In-depth Information of 252 *mcr-1*-positive strains used in phylogenetic analysis

Table S5. Pairwise comparison of SNPs of 253 *Salmonella* isolates

Table S6. Characteristics of the 4 primary *mcr-1*-positive *Salmonella* Clades

Table S7. Pairwise comparison of SNPs of 106 isolates carrying *mcr-1*-positive IncHI2 plasmids in comparison with the pHNSHP45-2 plasmid (Acc. no: KU341381)

Supplemental figures

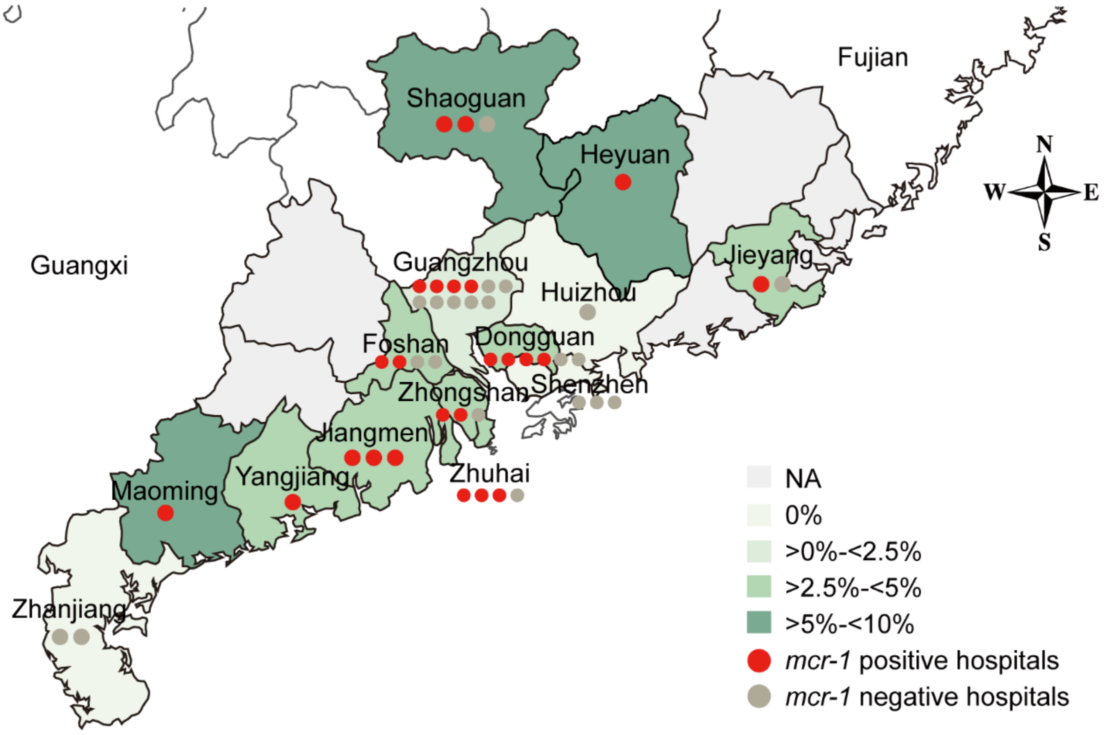

Figure S1. Geographic distribution and prevalence of *mcr-1*-positive *Salmonella* isolates from 46 hospitals in 14 cities, Guangdong, China

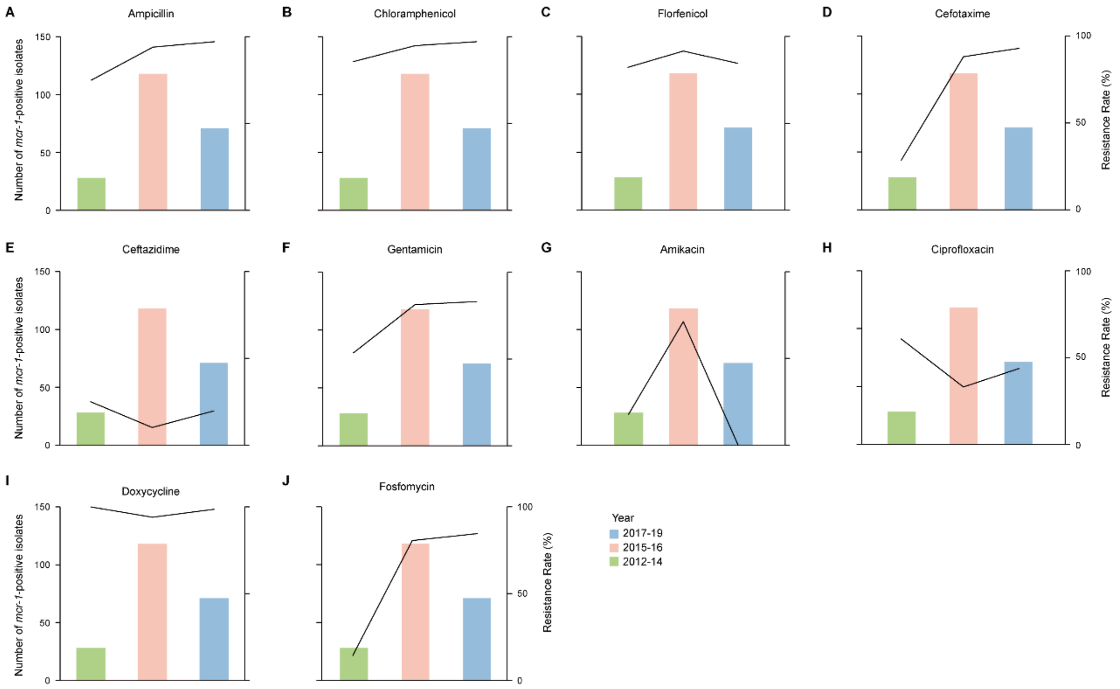

Figure S2. Change of antimicrobial susceptibility of *mcr-1*-positive *Salmonella* isolates over time

Left y-axis and histogram show the exact number of *mcr-1*-positive *Salmonella* isolates in each period. Right y-axis and line plot represent the proportion of isolates that are resistant to each antibiotic.

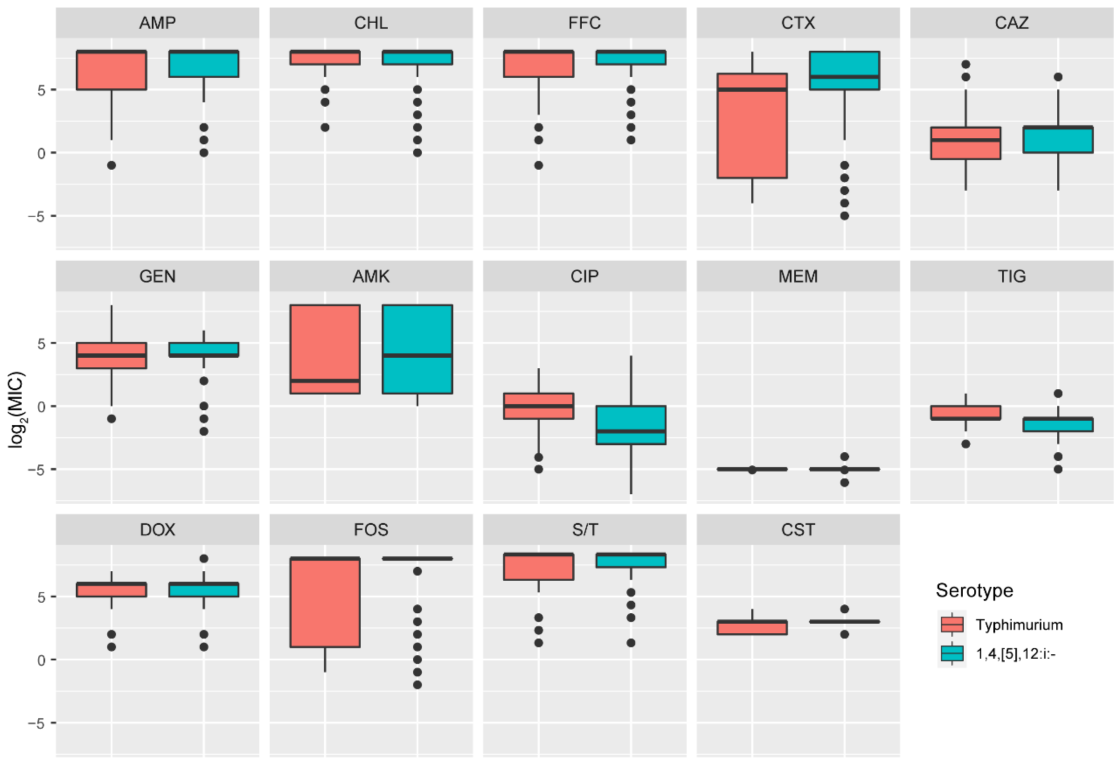

Figure S3. Distribution of MIC values for different antimicrobial agents for Typhimurium and its monophasic variant 1,4,[5],12:i:-

AMP: ampicillin, CHL: chloramphenicol, FFC: florfenicol, CTX: cefotaxime, CAZ: ceftazidime, GEN: gentamicin, AMK: amikacin, CIP: ciprofloxacin, MEM: meropenem, TIG: tigecycline, DOX: doxycycline, FOS: fosfomycin, S/T: sulfamethoxazole/trimethoprim, CST: colistin.

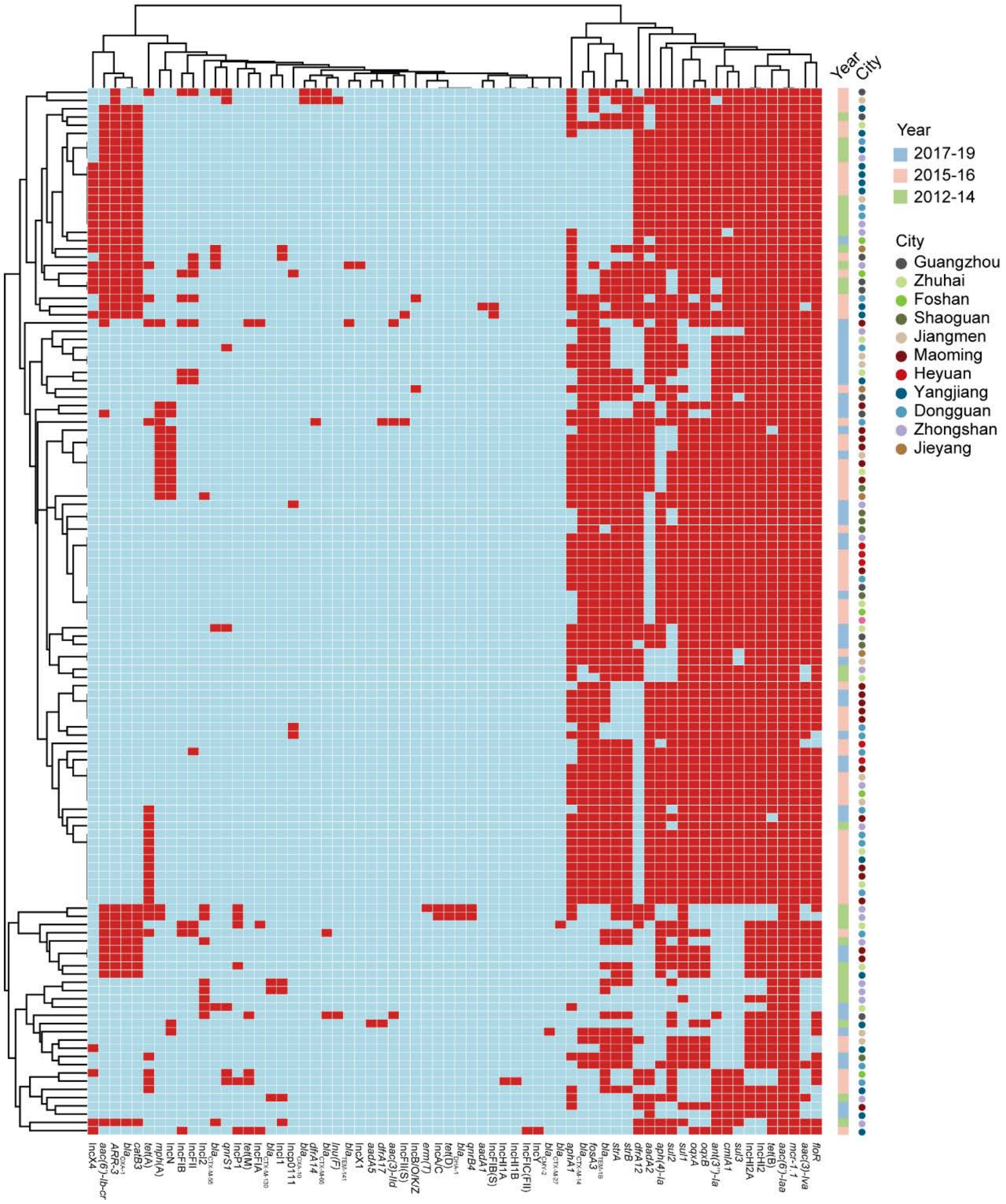

Figure S4. Distribution of plasmid Inc types and ARGs among the 127 *mcr-1*-positive isolates in present study

Red block denotes the presence of a plasmid Inc replicon or ARG. Columns are as follows: (i) the year of isolation; (ii) the city of isolation.

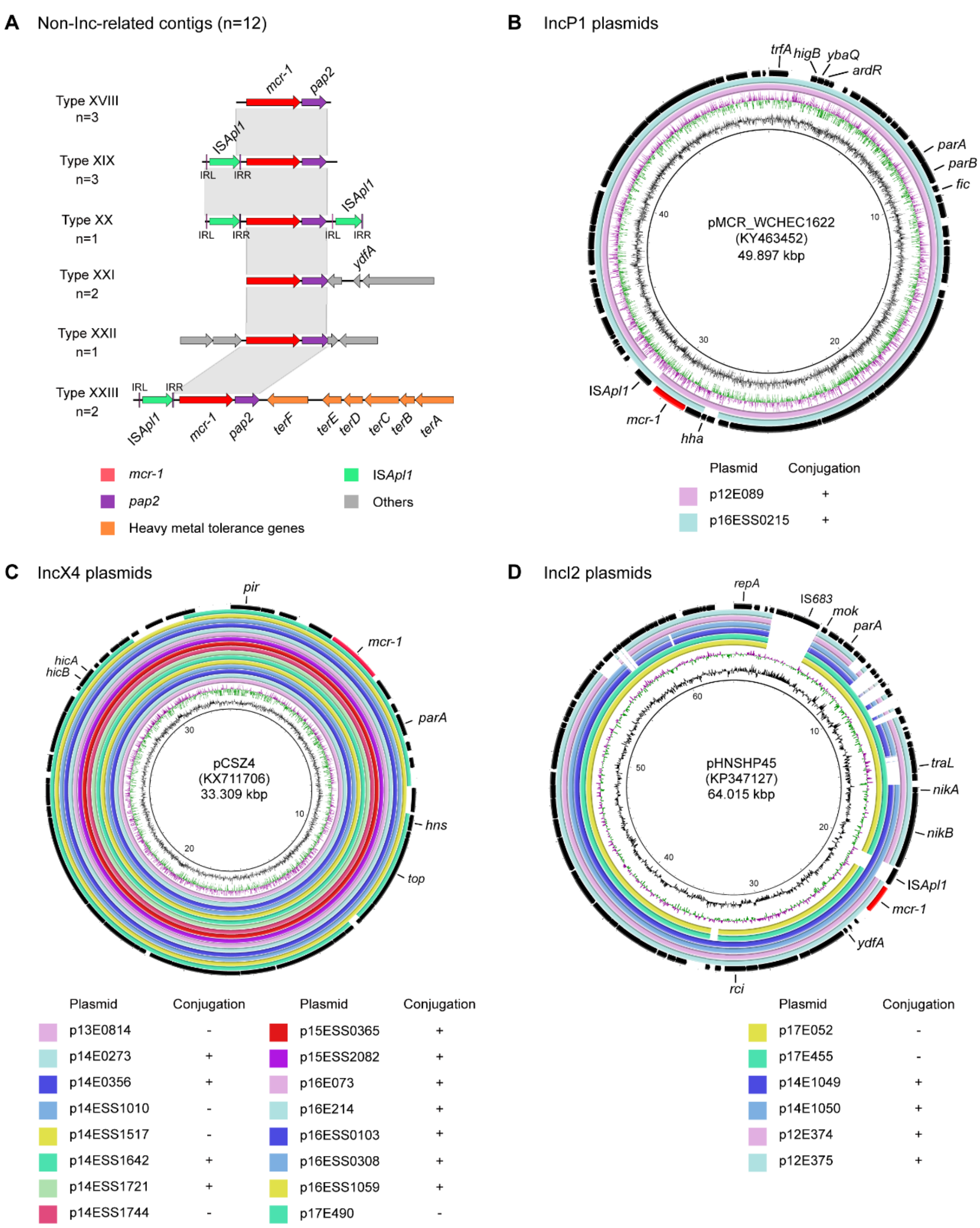

Figure S5. Sequence comparison between different *mcr-1*-carrying plasmids and contigs in this study

A. Genetic environments for non-Inc-related contigs containing *mcr-1* gene;

B. Genomic alignment of 2 *mcr-1*-harboring IncP1 plasmids with reference plasmid pMCR_WCHEC1622 (Acc. no: KY463452);

C. Genomic alignment of 16 *mcr-1*-harboring IncX4 plasmids with reference plasmid pCSZ4 (Acc. no: KX711706);

D. Genomic alignment of 6 *mcr-1*-harboring IncI2 plasmids with reference plasmid pHNSHP45 (Acc. no: KP347127).

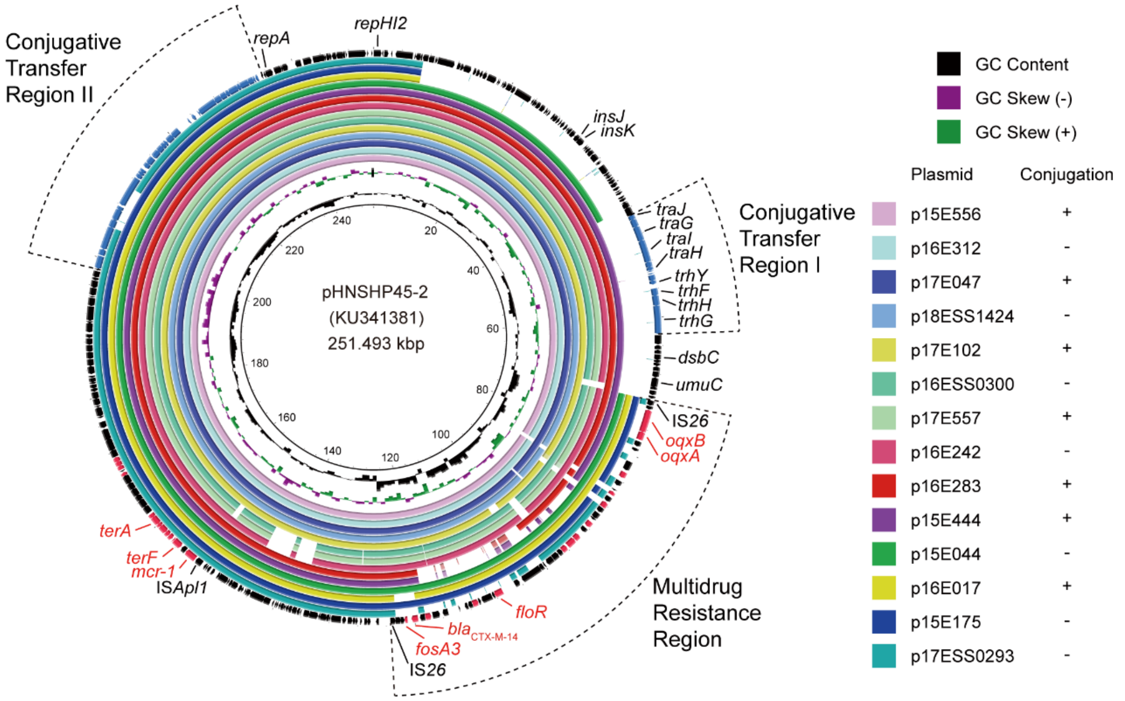

Figure S6. Circular alignments of reference plasmid pHNSHP45-2 (Acc. no: KU341381) with 14 selected complete IncHI2 plasmids from this study

The ARGs are indicated in red at the outer ring.

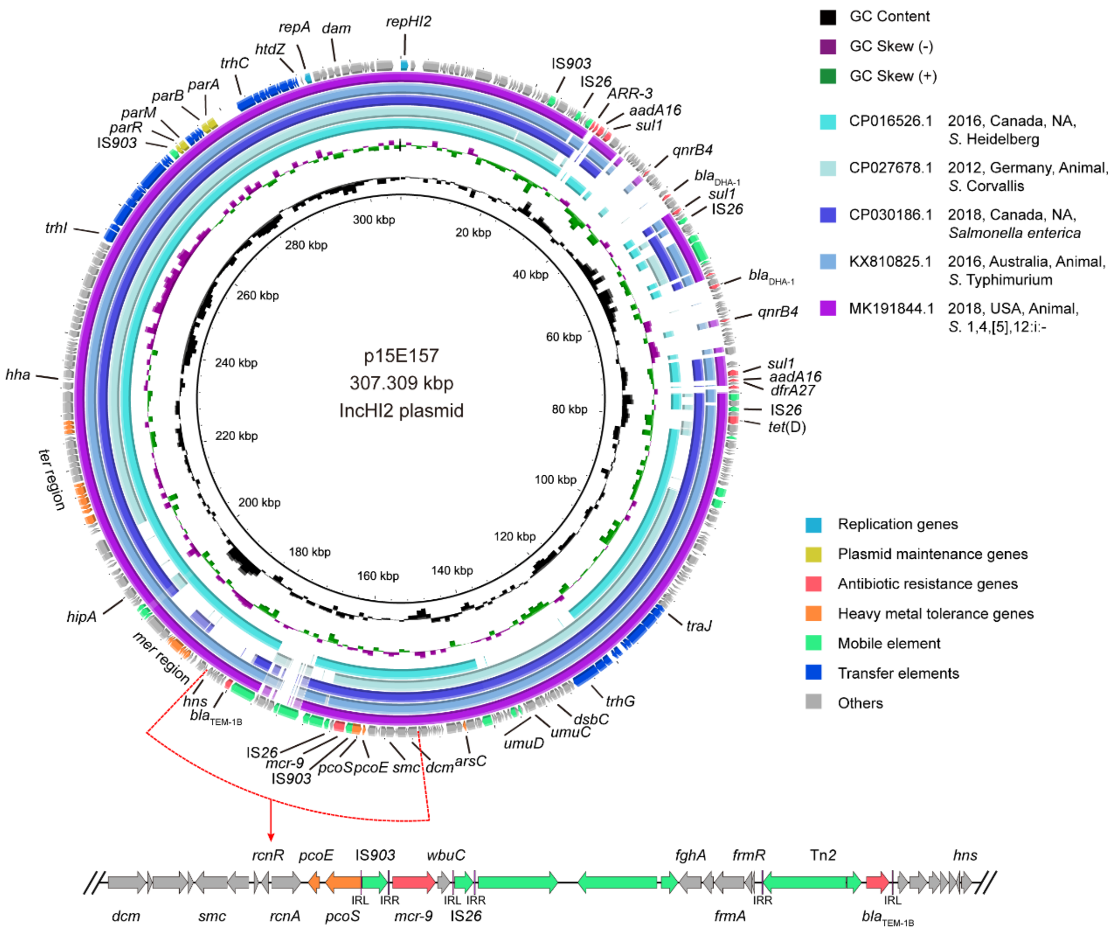

Figure S7. Genetic characterization of IncHI2-type *mcr-9*-harboring plasmids

Circular comparison between *mcr-9*-bearing IncHI2 plasmids found in this study and in the online database. The *mcr-9*-bearing IncHI2 plasmid p15E157 was used as the reference in the outermost ring.

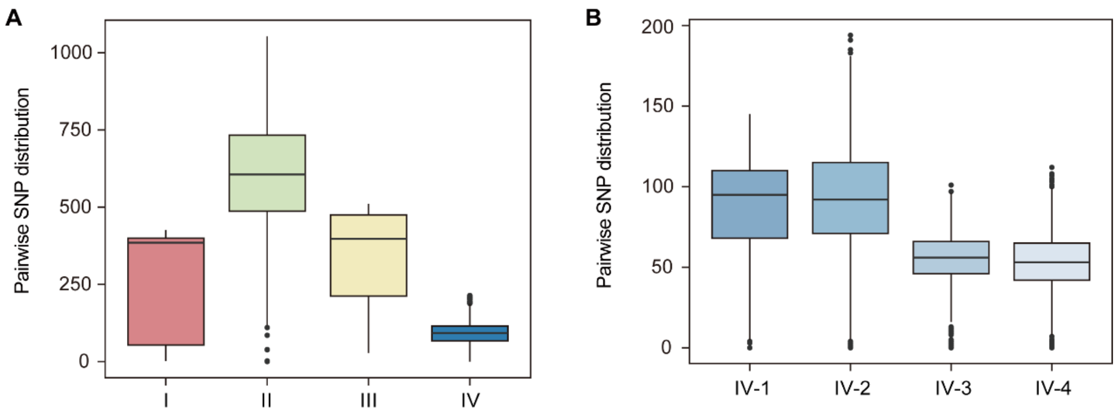

Figure S8. Core-genome pairwise SNP comparisons within each major BAPs lineage

A. Pairwise distribution of SNPs within Clade I to IV;

B. Pairwise distribution of SNPs within Subclade IV-1 to IV-4.
